# Effects of a Mindfulness Intervention Comprising an App, Web-Based Workshops, and a Workbook on Perceived Stress Among Nurses and Nursing Trainees: Protocol for a Randomized Controlled Trial

**DOI:** 10.2196/37195

**Published:** 2022-08-02

**Authors:** Simone Schönfeld, Ines Rathmer, Maren M Michaelsen, Cosima Hoetger, Miriam Onescheit, Silke Lange, Lena Werdecker, Tobias Esch

**Affiliations:** 1 Institute for Integrative Health Care and Health Promotion (IGVF) Faculty of Health/School of Medicine Witten/Herdecke University Witten Germany; 2 Interprofessional Graduate College in Integrative Medicine and Health Witten/Herdecke University Witten Germany

**Keywords:** nurses, nursing trainee, nursing student, acute care, inpatient, health promotion, mindfulness, mobile, web-based, stress, mobile phone

## Abstract

**Background:**

Previous research has found digitally supported mindfulness interventions to be effective when used for stress management among workers in high-stress occupations. Findings on digitally supported mindfulness interventions among nurses working in acute inpatient care settings are heterogeneous, lack long-term follow-up, and do not assess adherence and acceptability.

**Objective:**

This study aimed to investigate the effectiveness and efficacy of a digitally supported mindfulness intervention designed to improve health- and work-related outcomes among nurses and nursing trainees working in acute inpatient care settings.

**Methods:**

We will conduct a multicenter randomized controlled trial using a wait-list control group design. Randomization will be stratified by hospital and job status (nurse or nursing trainee). Recruitment will take place on the web and offline during the working hours of nurses and nursing trainees. The intervention group will receive a digitally supported mindfulness intervention, which will comprise an app, 2 web-based workshops, and a workbook, whereas the wait-list control group will be scheduled to receive the same intervention 14 weeks later. The 2 web-based workshops will be led by a certified mindfulness-based stress reduction trainer. Nurses will use the app and the workbook independently. Self-report web-based surveys will be conducted on the web at baseline, at 10 weeks after allocation, at 24 weeks after allocation, and at 38 weeks after allocation. Outcomes of interest will include perceived stress (primary outcome), health- and work-related variables, and variables related to adherence and acceptability of the digitally supported mindfulness intervention. We will perform intention-to-treat and per-protocol analyses.

**Results:**

Data collection will be completed by the beginning of August 2022. Data analyses will be completed by December 2022.

**Conclusions:**

Our study design, including long-term follow-up and the investigation of variables related to adherence and acceptability, will ensure rigorous evaluation of effectiveness and efficacy. Relative to costly in-person intervention efforts, this program may present a cost-effective and potentially highly scalable alternative. Findings regarding effectiveness, efficacy, adherence, and acceptability will inform stakeholders’ decisions regarding the implementation of similar interventions to promote the well-being of nurses and nursing trainees, which may, in turn, alleviate detrimental stress-related outcomes (eg, burnout) because of work-related demands.

**Trial Registration:**

German Clinical Trials Register DRKS00025997; https://tinyurl.com/433cas7u

**International Registered Report Identifier (IRRID):**

DERR1-10.2196/37195

## Introduction

### Need for Effective Stress Management Interventions for Nurses

The increasing psychological and physical burdens and high workloads of nurses and nursing trainees worldwide have been raising concerns for some time [1,2]. Nurses and nursing trainees in acute inpatient care settings are disproportionately affected by burnout and mental illnesses. Nursing trainees also experience high stress levels, with 34% reporting depressive symptoms before the COVID-19 pandemic [3]. Working in acute inpatient care settings has grown to be increasingly demanding throughout the pandemic; findings from a meta-analysis revealed that 30% [2] to 43% [1] of nurses experience work-related stress. The stress of health care professionals may diminish the quality of care provided to patients and can negatively affect other system-relevant factors [4-7]. Stressful work conditions may also decrease the willingness of nurses to continue working within their profession, which in turn facilitates nursing staff shortages in several countries [8], including Germany [2].

Currently, knowledge about effective stress management interventions to reduce these global challenges is limited. The available research on the effectiveness of stress management interventions for nurses has limitations, including small sample sizes limiting the validity and generalizability of the results and a lack of long-term follow-up [9-11]. More research is needed to examine which interventions result in positive outcomes and, more importantly, the conditions on which intervention effectiveness depends (eg, care setting and acceptability). Therefore, this study distinguishes between efficacy and effectiveness. Efficacy refers to the observed intervention effect when the protocol is followed, whereas effectiveness refers to the effect observed for the entire sample, regardless of protocol adherence.

### Requirements for Interventions in Acute Inpatient Care Settings

Stress management interventions should be designed to meet the needs of the target population to effectively decrease stress. A current review showed that stressors and work demands vary across care settings (eg, geriatric care and emergency hospitals) [12]; thus, the integrability of stress management interventions differs across settings and, consequently, so does the added value for all health care professionals to whom the intervention is offered.

Acute inpatient care settings are characterized by the treatment of sudden, urgent, and often life-threatening injuries and illnesses requiring rapid treatment; for example, such treatments include emergency medicine, trauma care, acute care, and critical care [13]. Acute inpatient care settings are marked by an increased risk of immediate involvement in traumatic events, such as experiencing the inability to save a patient’s life or feeling overextended because of an inadequate nurse-to-patient ratio [14].

Implementing stress management interventions for nurses working in acute inpatient care settings is difficult because of nurses’ demanding work schedules such as changing shifts and difficulties in scheduling breaks in advance [8]. In addition, COVID-19–related safety precautions limit the options of implementing in-person interventions.

In conclusion, appropriate stress management interventions for this target group should help in coping with challenging emotions, be effective in decreasing stress in the long term, and take into account nurses’ working conditions when it comes to integrability in the daily working routine.

### Digitally Supported Mindfulness Interventions

Digitally supported interventions offer a variety of user-modifiable content. They present a flexible, user-friendly, and cost-effective stress management option as users can engage in stress management practices at a time and place of their convenience.

An effective tool for stress management is mindfulness, especially for individuals with psychological distress [15,16]. Mindfulness has been operationalized as “the awareness that emerges through paying attention on purpose, in the present moment, and nonjudgmentally to the unfolding of experience moment by moment” [17]. The most thoroughly researched mindfulness intervention is mindfulness-based stress reduction (MBSR), which is a standardized 8-week course aimed at reducing stress [17]. Mindfulness interventions have been found to be effective for stress reduction and coping with challenging emotions in a variety of populations [15], even if the intervention was digitally supported [18,19]. Findings from systematic reviews and meta-analyses of randomized controlled trials (RCTs) focusing on nonclinical adult populations suggest that digitally supported mindfulness interventions could effectively improve perceived stress [18,19].

The results from the few available RCTs on digitally supported mindfulness interventions among nurses working in acute inpatient care settings are heterogeneous, do not include long-term follow-up (maximum 4 months), and have rarely been conducted in Europe (except for the study by Fiol-DeRoque et al [16]) [20-23]. Two studies showed significant reductions in anxiety and stress, as well as an increase in work satisfaction, following participation in digitally supported mindfulness interventions after 3 months [20-22]. However, 3 studies found no significant between-group differences in their outcomes [16,22,23]. They had an observation time of 1 month and 1 to 4 months [22].

As regular (preferably daily) practice is considered a prerequisite for efficacy [18], heterogeneous results may stem from a lack of acceptance of digitally supported mindfulness interventions, which leads to insufficient or infrequent meditation practice. High dropout rates present a prevalent problem in digitally supported mindfulness intervention research, with dropout rates ranging from approximately 25% [21] to approximately 48% [20]. Nonadherence in intervention instructions or dropout may occur when demanding work schedules and time constraints prevent participants from engaging in meditation practice to a degree that is necessary to achieve desirable outcomes. International differences in health care systems and cultural norms may also influence interest and participation in specific interventions on a regular basis [15].

To decrease dropouts and increase meditation practice, variables related to adherence and acceptability should be investigated [22]. This study distinguishes between adherence and acceptability. Adherence refers to the degree to which participants follow a treatment protocol [24], such as instructions regarding the frequency of meditation practice. Acceptability refers to the factors influencing digitally supported mindfulness intervention use.

In addition, steps should be taken to provide participants with engaging interventions to further treatment adherence and limit dropout risk, which could be achieved by adding user-engaging elements such as web-based workshops and workbooks. Mindfulness workbooks with writing exercises can be effective in stress reduction [25,26] and can be easily used by individuals with limited technological affinity. Previous research suggests that social interactions may enhance the efficacy of digitally supported interventions [15]; for example, studies on individuals with depression found digital interventions to be significantly more effective in reducing depressive symptoms when the intervention was guided by a mental health professional [26,27]. In addition, providing participants with the opportunity to share their experience with the intervention app in a web-based workshop setting could limit dropout risk [20].

Overall, the results on the effectiveness and efficacy of digitally supported mindfulness interventions in nurses working in acute inpatient care settings are rare, which limits the generalizability to different cultural and health care system contexts. Furthermore, the results of these studies are mixed. An intervention period of 3 months has been deemed effective in reducing stress [20,21]. However, the long-term effects (access to intervention for >4 months) of digitally supported mindfulness interventions have not yet been investigated. Importantly, to understand the mixed results in effectiveness, factors associated with adherence and acceptability in nurses deserve further investigation [9].

### Aims and Research Questions

Short-term (10-week after intervention start) and long-term efficacy and effectiveness (24-week and 38-week after intervention start) of a digitally supported mindfulness intervention for nurses and nursing trainees will be investigated. As the efficacy and effectiveness of an intervention are likely influenced by participants’ degree of adherence to the instructions and acceptability, we will investigate variables that may increase or decrease the likelihood of participant adherence and acceptability. The research questions are as follows:

1. Relative to a wait-list control group (WCG), does access to a digitally supported mindfulness intervention improve subjective health- and work-related outcomes among nurses and nursing trainees in acute inpatient care settings randomized to the intervention group (IG) at 10 weeks after allocation?
2. Relative to scores observed at baseline, does access to a digitally supported mindfulness intervention improve subjective health- and work-related outcomes among nurses and nursing trainees in acute inpatient care settings at 10, 24, and 38 weeks after intervention start? To what degree do app-based minutes of meditation predict each of our outcomes?
3. Which variables are associated with adherence to a digitally supported mindfulness intervention at 10, 24, and 38 weeks after the intervention starts among nurses and nursing trainees in acute inpatient care settings?
4. Which variables are associated with the acceptability of a digitally supported mindfulness intervention at 10, 24, and 38 weeks after the intervention starts among nurses and nursing trainees in acute inpatient care settings?

## Methods

### Design

We will conduct a multicenter RCT with a WCG design using individual-level randomization, stratified by the hospital or nursing school. The WCG will receive the digitally supported mindfulness intervention 14 weeks after allocation.

#### Study Setting

This study will be conducted at 4 hospitals and hospital-associated nursing schools in North Rhine-Westphalia, Germany. The number of nurses employed at each institution ranges from 40 to 1400. Two of the hospitals are acute care hospitals with emergency departments, whereas the other 2 hospitals specialize in pneumonology and cardiac surgery. The 2 nursing schools currently have a total of 280 and 380 enrolled nursing trainees.

#### Eligibility Criteria

Eligible participants were aged ≥18 years, self-identified as nurses or nursing trainees, and reported being employed full-time or part-time at one of the participating hospitals at the time of data collection. Finally, the participants were required to have access to a smartphone. Individuals who did not report employment at one of the participating hospitals or did not self-identify as nurses or nursing trainees were not eligible to participate.

#### Recruitment

Recruitment took place between August and October 2021 during hospital staff meetings, as well as during web-based meetings among project partners, which were attended by nurses. In addition, participants were recruited via weekly newsletters, handbills, and flyers handed out at participating hospitals and nursing schools.

Eligible nurses and nursing trainees interested in participating in the study were able to register via a website and were emailed the link to the time point 0 (T0) survey (baseline survey). Upon obtaining informed consent, participants were prompted to fill out the web-based survey. Information regarding the study design was not disclosed to the participants.

Informed consent was obtained from all participants before the start of each web-based survey. Informed consent materials are currently available only in German (Multimedia Appendix 1). To prevent individuals from participating more than once, we checked for duplicates in the first and last names and email addresses during registration. Each registered person received personalized invitation letters via email with an individual access key to the survey (token).

Incentives for study registration and survey participation were available at the discretion of each hospital’s management, thus varying across locations. A raffle was initiated by the intervention provider at one of the hospitals to incentivize staff participation in the surveys. Incentives included 15 gift cards €15 (US $15.8) each for various shopping websites for the first 2 surveys, whereas the last survey was incentivized by the opportunity to win 1 of the 10 gift cards €30 (US $31.6) each. At some participating hospitals, participants were allowed to participate during work hours, whereas others required their staff to partake during their personal time.

#### Participant Timeline

The study will be conducted over a 10-month period (Table 1). A total of 4 surveys will be conducted 3 months apart from one another. Participants will be allowed 2.5 weeks to complete each survey. All participants randomized to the IG will receive access to the digitally supported mindfulness intervention 2 weeks after T0 data collection. All participants randomized to the WCG will receive access to the digitally supported mindfulness intervention 2 weeks after time point 1 [T1] data collection (14 weeks after the IG has been given access). Data collection for both groups will take place at 10, 24, and 38 weeks after allocation. In other words, data collection for each group will take place at 10 weeks (T1 for the IG and time point 2 [T2] for the WCG) and 24 weeks (T2 for the IG and time point 3 [T3] for the WCG) after intervention start. For individuals randomized to the IG, data will be collected at 38 weeks after the intervention starts (T3).

**Table 1:** Schedule of enrollment, interventions, and assessments for the intervention group (IG) and the wait-list control group (WCG).

		Enrollment	Baseline	Random allocation	After allocation			
			T0^a^	Intervention start IG	T1^b^	Intervention Start WCG^c^	T2^d^	T3^e^
**Enrollment**								
	Eligibility screen	✓						
	Informed consent	✓	✓		✓		✓	✓
	Allocation			✓				
**Intervention^f^**								
	For IG			✓	✓	✓	✓	✓
	For WCG^c^					✓	✓	✓
**Assessments**								
	Participant characteristics^g^		✓		✓^h^		✓^h^	✓^h^
	Primary outcome		✓		✓		✓	✓
	Secondary outcomes		✓		✓		✓	✓
	Adherence		✓		✓ (only IG)		✓	✓
	Acceptability		✓		✓ (only IG)		✓	✓

^a^T0: time point 0.

^b^T1: time point 1.

^c^14 weeks after IG.

^d^T2: time point 2.

^e^T3: time point 3.

^f^Digitally supported mindfulness intervention.

^g^Sociodemographic and job-related variables.

^h^Participant information will be updated when applicable.

#### Assignment of Interventions: Sequence Generation

Only individuals completing the baseline survey T0 were randomized to one of the study conditions. Randomization and allocation were performed by a third blinded research team member not involved in data analyses. Randomization was stratified by hospital and nursing school and job status (nurse or nursing trainee; 8 strata). Randomization was conducted using the website [28] (computer-generated random numbers), which generates a string of numbers comprising 1 (IG) and 2 (WCG) in random order. The length of the list was predefined and dependent on the number of participants per strata. The third blinded researcher combined each list of random numbers with the strata list of participants, thus randomizing each individual to either the intervention or control group. After the allocation, a research team member sent participants an email containing an access code for the app, links for web-based workshops, and a pickup location for workbooks.

#### Intervention

**Figure 1:**
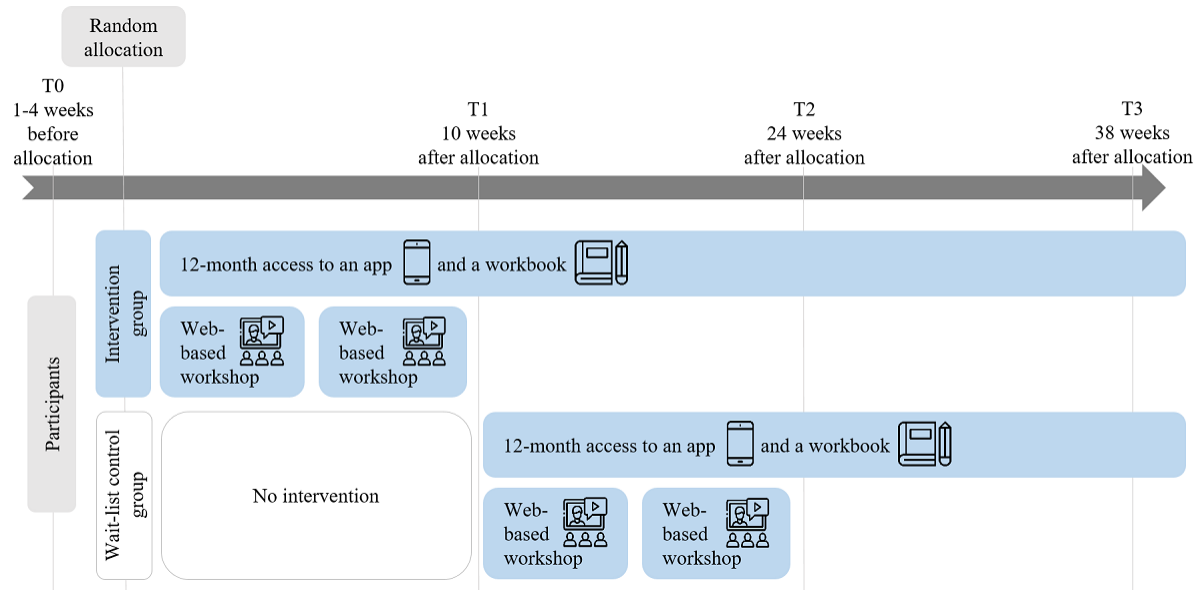
Timeline of access to the intervention in the multicenter study along four time points: time point 0 (T0), time point 1 (T1), time point 2 (T2), time point 3 (T3; icons: Flaticon [29]).

The intervention will be initiated by email. The email will contain the app access code, workbook pickup location at each hospital, and a list of proposed dates for web-based workshops. Participants will be given instructions on how to download the app from the app store and activate the code. The access code will allow access to the full app version, including meditations and education courses tailored to nurses for a 12-month period (Figure 1 [29]). The intervention will be free of charge for the participants.

The standardized multimodal stress management intervention comprises 3 components (an app, 2 web-based workshops, and a workbook). The web-based workshops and the workbooks are supplemental to daily app-based meditation. In-person interaction will occur only during the web-based workshops when participants will interact with the MBSR trainer and other group members. In case technical difficulties are encountered, information technology support will be available to the participants.

The app contains >900 meditation exercises and educational content on mindfulness and meditation. The app is available in German, French, Dutch, and English. In addition, the app features 3 content areas tailored specifically to nurses, which were developed by researchers in the nursing field (Table 2).

**Table 2:** App-based nurse-specific care content.

Name			Number of meditations per course^a^		Duration per meditation (minutes)^a^		Content
**Specific courses for nurses**							
	Mindfulness in Everyday Care	7		7-10		Introduction to mindfulness meditation and other relaxation techniques; the course content is derived from scientifically based courses such as the Kabat-Zinn [17] mindfulness-based stress reduction, mindful self-compassion, and reflection and relaxation techniques such as autogenic training and progressive muscle relaxation, which can be used daily.	
	Resilience in Care 1	7		7-9		During the first week, participants’ personal resources (eg, ability to respond mindfully, self-acceptance, self-efficacy) will be strengthened using mindfulness and self-compassion exercises.	
	Resilience in Care 2	7		7-9		During the second week, participants will learn to use visualization and gratitude and reflection exercises to identify external resources and will learn strategies on how to access these resources in their daily life.	
	Resilience in Care 3	7		7-10		During the third week, participants will learn how to cope with stressful situations in which one’s resources prove to be insufficient; in addition, participants will be taught stress management exercises specifically designed to decrease rumination and promote relaxation.	
	Resilience in Care 4	7		7-10		The fourth and final week of the course will teach participants coping skills to navigate job-related challenges frequently encountered by nurses working in hospital settings; coping skills include setting boundaries and practicing effective communication and compassion toward others and oneself.	
**Short situation-specific meditations in everyday care**							
	Everyday Care	14		3-9		Designed to help nurses integrate mindfulness into their daily work activities; for example, meditations can be used when arriving at the hospital while walking down the hallway, or at the end of the workday; these short meditations are designed to decrease rumination, encourage nurses to take a break to focus on self-compassion, increase well-being, and help nurses cope with negative emotions.	

^a^Meditation=one audio file.

Nurses and nursing trainees will be invited to participate in 2 web-based workshops led by a certified MBSR trainer. The web-based workshops (approximately 60 minutes) will take place 1-2 weeks after app access has been granted. Up to 50 participants can take part in each web-based workshop. Per workshop, there will be 4 appointment options. The dates will be scheduled to accommodate individuals’ work schedules, and appointments will be offered before and after shifts. Course content on mindfulness and resilience, as well as app-based and non–app-based meditation exercises, will be presented along with advice on how to implement stress management techniques at work. The second workshop will take place approximately 6 weeks after the first workshop. The learning objectives of the second course will be to engage participants with one another, facilitate the exchange of ideas, clarify questions, reflect on relaxation behavior, and discuss motivation and strategies to increase the likelihood of daily meditation.

To supplement the daily content of the 2 app-based courses (*Mindfulness in Everyday Care* and *Resilience in Care*), each participant will be given a paper copy of a mindfulness workbook containing information specifically tailored to nurses. Within the workbook, information regarding the concept of mindfulness and instructions on the use of the app-based courses will be provided. Course content will include self-compassion, resilience, interpersonal relationships, conflict resolution, and boundary setting. In addition, participants will be prompted to provide answers daily to questions including their most enjoyable moment of the day, stressful experience, perceived stress level, mood, quality of sleep, level of relaxation, and quality of social support received.

Participants will be instructed to meditate daily via the app, web-based workshops, and workbook. No standardized prompts or reminders to meditate will be provided, and participants will be able to choose the frequency and duration of their meditation freely. Participants will have the option to choose whether to receive a weekly email newsletter from the intervention provider.

App development started in 2014. Since then, the app has been one of the most frequently downloaded meditation applications in Germany; health insurance companies cover the costs associated with gaining access to specific app-based prevention courses that are otherwise only available via the premium version of the app. There is only one previous peer-reviewed evaluation among office workers indicating significant improvements in mindfulness, work engagement, job satisfaction, emotional exhaustion, emotional intelligence, innovation and creativity, and self-efficacy after using the app for 14 days [30].

No substantial revisions to the app are planned, and the anticipated app updates are limited to minor bug fixes. The present intervention will use the app versions available in the Apple App Store and Google Play Store. No quality assurance methods have been planned to ensure the accuracy and quality of the information provided by the intervention provider.

#### Data Collection, Management, and Monitoring

Data will be collected via a web-based data collection tool (*LimeSurvey*; LimeSurvey GmbH [31]), provided by the study sponsor’s affiliated university. Participants will be allowed to skip questions if they experience discomfort. At the end of each survey, the participants may enter feedback or additional comments into a text box. Participants will receive reminder emails prompting them to complete the surveys. Data from randomized noncompleters will be used to calculate the loss to follow-up rates.

Data management will take place at the site of the study sponsor. The research team members will be responsible for data entry, coding, security, and storage. Range checks for data values will be conducted, and additional steps will be taken to ensure data quality; data checks will be performed by 2 research team members who will also double check whether the coding has been performed correctly. Data will be stored on a safe university-based network location that will only be accessed by authorized research staff.

The data monitoring committee will comprise the team members of the study sponsor. There will be a regular correspondence between the data collection site and the principal investigator to ensure adherence to the protocol, which has been approved by the university-based institutional review board (IRB). The study funder will reserve the right to terminate the study at any point. Adverse events will be reported to the IRB. Documentation of such events will be stored on the university’s safe network location. No interim analyses are planned.

#### Outcomes

The German version of each questionnaire has been previously validated. All outcomes of interest consist of continuous variables based on self-report data. Our primary outcome will be perceived stress measured using the 10-item Perceived Stress Scale (PSS-10) [32]. Secondary outcomes will include sense of happiness (Likert scale) [33], life satisfaction (L-1) [34], mindfulness (Five Facet Mindfulness Questionnaire) [35], well-being (World Health Organization-Five Well-Being Index) [36], self-care (Hamburg Self-Care Questionnaire; only pacing scale) [37], pain intensity (numerical rating scale 0-10) [38], work-related sense of coherence questionnaire [39], burnout (Copenhagen Burnout Inventory; work-related burnout scale and client-related burnout scale) [40], job satisfaction (Warr-Cook-Wall Scale [41], modified by Cooper et al [42]), and work engagement (Utrecht Work Engagement Scale) [43].

#### Other Variables

##### Participants’ Characteristics

Sociodemographic variables will include age (in years), gender (male, female, or diverse), relationship status (categorical variable), children (yes or no), previous meditation experience (yes or no), and participation in other studies to promote health (yes or no).

We will assess for job-related variables. We will assess participants’ job status (nurse or nursing trainee) and work status (part-time or full-time); if part-time, we will assess the number of work hours per week (per employment contract), number of hours worked per week during the past 4 weeks (including overtime), patient contact during the past 4 weeks (scale 0%-100%), current area of care (eg, internal medicine, surgery, multiple possible answers), duration of employment at the current workplace (in years), job-related tasks that involve caring for patients with COVID-19 more than half of the time (yes or no), working in a hospital with incentives for study participation at T0 (yes or no), and employer permission to participate in study activities during work hours (yes or no). We will assess the following job-related variables for only nurses: qualifications beyond training (eg, additional qualifications, bachelor’s degree, or master’s degree) and years of work experience. We will assess the following job-related variables for nursing trainees only: focus of training (general nursing, pediatric nursing, or care of older adults) and year of training.

##### Adherence

Adherence to the instructions for using the digitally supported mindfulness intervention will be operationalized as daily meditation. The frequency of meditation practice (app-based and non–app-based meditation) will be recorded based on the following answer options: several times per day, daily, 4 to 6 times per week, 1 to 3 times per week, less than 1 time per week, or never.

##### Acceptability

The acceptability of the digitally supported mindfulness intervention will be operationalized as perceived usefulness through 4 separate questions such as “How useful do you rate the [app/online workshop/workbook/all in all]?” We will use a 5-point Likert scale (1=not at all applicable to 5=very applicable). We will assess which of the nursing-specific courses were helpful (listing of the names of the nursing-specific courses; multiple answers possible).

##### Intensity of Intervention Use

App use will be measured through previous experience with the app (“yes, I have tried out the app”; “yes, I use the app regularly”; or “no, I have never used the app”), previous experience with the app before code activation (“yes, I have tried out the app”; “yes, I use the app regularly”; or “no, I have never used the app”), date of access code activation (yes [date of activation] or no), total amount of app-based meditation (in minutes), total number of app-based meditation exercises, subjective assessment of app use frequency (several times a day, daily, 4-6 times per week, 1-3 times per week, less than once a week, or never), time of use (in my free time, before my shift, during my shift, during my breaks on shift, or after my shift), and use of the app-based nursing-specific courses (list of nursing-specific courses; selection of multiple answers).

Participation in the 2 user-engaging components (web-based workshop and workbook) will be assessed using a binary variable (yes or no). Frequency of workbook use will be assessed using the following answer options: several times a day, daily, 4 to 6 times per week, 1 to 3 times per week, less than once per week, or never. Finally, we will assess for non–app-based meditation (yes or no).

#### Sample Size

On the basis of the available literature on interventions similar to ours, we assumed a medium effect size (Cohen *d*=−0.67) in reducing stress measured by PSS-10 [44]. The sample size calculation was conducted for a 2-tailed *t* test with a significance level of α=.05 and a power of β=.80 (comparison between the IG and the WCG). Sample size calculations using the G*Power tool (Düsseldorf University) suggested that 72 participants (36 in the IG and 36 in the WCG) are needed to detect an effect size of 0.67. Assuming a dropout rate of 20%, 18 additional participants were required (45 per group).

#### Statistical Methods

##### Overview

Analyses will be conducted using *SPSS Statistics* (IBM)*.* For tests involving our primary outcome variable, we will set an α level of .05. For all other analyses, to reduce α-error inflation, we will interpret the obtained *P* values descriptively, meaning all other analyses will be exploratory and are designed to observe trends and not to identify significance.

Patterns of missing data will be assessed, and adequate data imputation techniques such as multiple imputation with *SPSS Statistics* (IBM) will be applied. Before doing so, we will assess the percentage of missing data. We will also use Little's Missing Completely At Random test to determine patterns of missing data (ie, missing completely at random vs not missing at random).

We will reverse code 3 outcomes [32,38,40] so that a higher score indicates health improvement. For example, the scale assessing burnout [40] is designed to indicate that a score of 1 indicates a rather minuscule risk of burnout, whereas a score of 100 indicates the highest risk of burnout, meaning that greater scores indicate a worsening of symptoms. After recoding this variable, greater scores will reflect a lower risk of burnout (ie, an improvement in symptoms).

Effectiveness will be evaluated using an intention-to-treat (ITT) approach; all randomized participants will be included in our analyses [45].

Efficacy will be evaluated using a per-protocol (PP) approach; only study completers who report adherence will be included in our analyses.

Several analyses will be used to address the research questions. To ensure that our randomization has worked as intended, we will assess for between-group differences for relevant participant characteristics and, if needed, adjust the following analyses. For research questions 2, 3, and 4, we will create a pooled data set merging the data of the IG and the WCG. We will merge the data of the 2 groups so that IG T0 data will be combined with WCG T1 data, and IG T1 data will be combined with WCG T2 data, as described in Textbox 1. For research questions 3 and 4, relationships between variables will be examined using correlation analyses (Pearson correlation coefficient, eta quotient, and chi-square tests). If violations of the assumptions associated with any of the analyses are detected, alternative tests will be used.

**Data pooling procedure.**

**Newly pooled data set and data included**

- Participant data baseline
  Cases of the intervention group at time point 0
  Cases of the wait-list control group at time point 1
- Participant data 10 weeks after intervention start
  Cases of the intervention group at time point 1
  Cases of the wait-list control group at time point 2
- Participant data 24 weeks after intervention start
  Cases of the intervention group at time point 2
  Cases of the wait-list control group at time point 3

##### Research Question 1

We will present the ITT sample’s baseline characteristics and test for detectable between-group differences. For continuous variables, mean, SD, median, minimum, and maximum values will be calculated. For categorical variables, frequencies (absolute and percentages) will be calculated.

To evaluate the effectiveness and efficacy of the 10-week access, we will create one difference score per group for each of our outcomes assessed at T0 and T1. Using a 2-sample *t* test, we will test whether significant differences exist between the 2 difference scores in the IG and WCG. We will perform this for the ITT and PP sample. If *P*<.05, Cohen *d* will be calculated.

##### Research Question 2

To evaluate long-term effectiveness and efficacy, we will use the pooled data set. To analyze the effect of time on our variables of interest, a repeated-measures ANOVA will be conducted for each of our outcomes. As only participants randomized to the IG will be assessed at 38 weeks after allocation, long-term efficacy within this group will be examined using an additional repeated-measures ANOVA.

Using a PP approach, we will examine the relationship between *app-based minutes of meditation* and each previously calculated difference score using the pooled data set. Linear regression will be performed to examine the relationship between the minutes of meditation and each outcome. The baseline scores of primary and secondary outcomes will be entered into the model as additional explanatory variables to control for individual differences before the start of the intervention.

##### Research Question 3

Using the pooled data set, to determine which variables are associated with adherence at 10 weeks and 24 weeks after intervention start, we will examine the correlations between the variable *frequency of meditation practice* and participants’ baseline characteristics, acceptability, and intensity of intervention use using the ITT sample. To assess adherence at 38 weeks after the intervention, analyses will be conducted using the ITT data set of the IG.

##### Research Question 4

Using the pooled data set, to determine which variables are associated with acceptability at 10 weeks after the intervention start and 24 weeks after the intervention start, we will examine the correlations between the variable of *perceived usefulness* (acceptability) and participants’ baseline characteristics and intensity of intervention use using ITT sample. To assess acceptability at 38 weeks after the intervention, analyses will be conducted using the ITT data set of the IG.

##### Sensitivity Analyses

To determine the reliability of the analyses for research questions 1 and 2, we will conduct sensitivity analyses for our primary and secondary outcomes. We will include only complete cases (complete data for T0, T1_,_ T2, and T3).

### Ethical Considerations

#### Research Ethics Approval and Amendments

The IRB approved this study in July 2021 (S-53/2021). If and when applicable, amendments will be submitted to the IRB. After the study is completed, all nurses employed at the study sites will be given free 12-month access to the intervention.

#### Confidentiality and Access to Data

Personal information about potential and enrolled participants will be collected only by members of the research team and cannot be accessed by other individuals. Personal information and survey data will be pseudonymized using an identification number (token). Only authorized study personnel will have access to any of the data associated with this study. The study funder reserves the right to share the anonymized data with other parties.

## Results

Data collection at T0 was conducted from the end of September 2021 until October 2021. At the end of October 2021, a total of 79 individuals were randomized to either the IG (40/79, 51%) or WCG (39/79, 49%). All data collection will be completed by the beginning of August 2022. Data analyses will begin after data collection and will be completed by December 2022.

## Discussion

Mixed results have been found for digitally supported mindfulness interventions for nurses working in acute inpatient care settings [16,20-23], highlighting the need for planned research involving long-term follow-ups and analyses of factors of adherence and acceptability-related variables.

### Expected Results

Our study design, involving long-term follow-up, a standardized intervention, and our planned investigation variables related to adherence and acceptability, will allow us to rigorously evaluate the effectiveness and efficacy of a digitally supported mindfulness intervention among nurses and nursing trainees working in acute inpatient care settings. The findings of our study will provide valuable information regarding the design and implementation of future stress management interventions for nurses and nursing trainees in acute inpatient care settings.

On the one hand, the digital nature of this intervention allows for the accommodation of nurses’ busy work schedules, thus increasing the likelihood of ongoing meditation practice and resulting in higher effectiveness. Given their user-engaging design, the workbook and web-based workshop components may further contribute to the participants’ maintained interest in digitally supported mindfulness intervention use. On the other hand, previous research has revealed that implementing health promotion efforts into a demanding daily work routine presents a challenge for nurses worldwide [46], and the unpredictable nature of COVID-19–related conditions likely exacerbates the challenge to implement new stress management habits.

### Comparison With Prior Work

For comparison with other studies, we will consider the method and context of the study. These include COVID-19–related working conditions, country, definition of ITT and PP, duration of the intervention, and previously identified variables related to adherence and acceptability.

### Generalizability

Results regarding effectiveness, efficacy, adherence, and acceptability may be generalizable to other health care professionals working in similar settings and nurses working in different care settings, as the app contains not only contents specifically tailored to nurses working in German inpatient care settings but also a variety of meditations and courses on various mindfulness topics (>900) independent of any specific context. By conducting a multicenter study, the generalizability may be further improved.

The generalizability of our results may be limited as COVID-19–related health care burden and hospitalization rates vary widely across countries and continents; stressors experienced by nurses and nursing trainees working in Germany may differ from stressors experienced by nurses working in other countries. In addition, nursing training requirements may differ across countries; for example, nursing trainees in Germany are required to gain hands-on work experience from the start of their training [47]. By receiving reminders to participate in the web-based surveys, participants are likely inadvertently reminded to interact with the app. As these reminders do not occur in real-world settings, the applicability of the findings from our RCT and others should be interpreted with caution.

### Strengths and Limitations

The study results will be reported in accordance with international documentation guidelines, including CONSORT-EHEALTH (Consolidated Standards of Reporting Trials of Electronic and Mobile Health Applications and Online Telehealth) statements [48,49] (Multimedia Appendix 2). We anticipate that our study will have high validity and reliability. In addition, an RCT allows for the identification of the causal effects of our intervention.

Our study will not include an active control group, which is a common limitation in similar studies. The study design was chosen as the primary aim of the study is to evaluate the intervention relative to no intervention, and WCGs are an ethical option to provide the intervention quickly to all participants [50]. A third experimental condition was discussed; however, we ultimately decided against this, given our small sample size.

Our WCG study design is associated with several limitations. This study design may overestimate intervention effects [50], as beneficial effects may occur among participants randomized to the IG simply because of the time spent away from work when participating in web-based workshops. These effects can be identified by including an active control group [10,51]. Furthermore, placebo effects and effects occurring because of participants’ expectations may occur with our study design. On the one hand, participants may experience some beneficial effects because of their anticipation of the upcoming intervention [52], which may be further increased by digitalization-specific expectations and participants’ trust in the study funder (a large health insurance company) [53]. On the other hand, previous research suggests that participants in WCGs do not improve until they receive the intervention [54].

Our study aims to investigate the effects of this intervention in its entirety. Our study design and analyses do not allow for conclusions regarding the intervention components that drive the identified effect or the proportion of the observed variance that is explained by each component. Some content commonly found in mindfulness interventions may lead to reduced stress [55]. However, several components could influence stress reduction among the participants in our study, irrespective of the mindfulness construct embedded in our intervention. First, the app contains educational content about stress management in general and features relaxation techniques such as the Jacobson progressive muscle relaxation. Second, the app prompts users to engage in self-reflection and mood monitoring and to read the educational content, which in itself can motivate the individual to engage in behavior change that may, in turn, facilitate stress reduction. Third, listening to nature sounds [56] or viewing visually appealing content may improve mood and enhance relaxation. Finally, the web-based workshops take place in group settings, and include social interaction with the trainer [15,55] and one’s peers may lead to stress reduction. However, we consider the variety of content provided to the users as a strength of the intervention, as users can choose the content most suitable and effective for them.

Selection bias because of the uneven distribution of confounders is usually not a cause for concern in large RCTs, as one can assume that latent and manifest variables are balanced equally across conditions. For smaller RCTs such as ours, there are steps that can be taken to reduce the risk of uneven distribution of latent or manifest variables across groups. To minimize the risk of confounding variables, we have used stratification for our randomization processes. Furthermore, single-blind randomization and allocation will be used to reduce selection bias. To ensure that our randomization has worked as intended, we will assess for between-group differences for relevant participant characteristics.

Detection bias may occur as blinding of participants is not possible, given the nature of the study. Although the study design is not communicated to the participants, the participants are to be likely aware of which group they have been randomized to. There may be a risk of social desirability as the effects will be evaluated based on subjective reports, including the frequency and intensity of digitally supported mindfulness intervention use, and no biological parameters will be collected. To reduce detection bias, all questions were tested among members of the target group before the start of the study, and all outcome measures were validated in previous studies. Evaluations of the psychometric properties of our primary outcome, the PSS-10, in different countries report a Cronbach α of .78 to .91, with good test-retest reliability [57]. PSS-10 scores correlate significantly with cortisol levels and constitute an objective measure of cumulative stress [58]. We will also seek to reduce the risk of social desirability by informing participants of the pseudonymization and anonymous publication of the data and by conducting data collection without in-person contact. In addition, we will not use participant data collected via the app but will allow participants to report data collected by the app. We refrained from tracking app use and the duration of use for privacy reasons. Not tracking app use may increase rapport, as participants will not feel monitored while using the app. Finally, the item with which we assessed gender may present a limitation as the German language uses the same term for sex assigned at birth and gender identity; thus, we cannot be certain whether participants reported their sex or their gender.

Performance bias will be reduced by using a standardized intervention; however, COVID-19–related differences may occur, as the WCG will receive the intervention at a later time.

Attrition bias may occur in individuals with little technical experience. By conducting a web-based survey, individuals with an affinity for technology are more likely to participate in the surveys on an ongoing basis. This restriction cannot be avoided because of pandemic-related regulations. To reduce bias through loss to follow-up (attrition bias), we will send reminder emails of survey participation, and participation in the survey will be incentivized by the intervention provider at one hospital. We will conduct ITT and PP analyses to reduce the risk of misinterpretation of results because of loss to follow-up.

### Conclusions

If effectiveness can be proven, the intervention outlined here may represent a standardized and cost-effective tool to reduce stress among a group of health care professionals experiencing increased stress. Given the strains that the COVID-19 pandemic has placed on health care workers, it is of utmost importance to provide this integral group of professionals with access to effective stress management interventions. Such interventions should be scalable to make them accessible to more individuals. Our results will inform stakeholders’ decisions regarding the design and implementation of future intervention efforts to enhance well-being among nurses and nursing trainees.

## Appendix

Multimedia Appendix 1 Used informed consent form in the German language.

Multimedia Appendix 2 CONSORT eHEALTH Checklist (V 1.6.1).

## Abbreviations

CONSORT-EHEALTH: Consolidated Standards of Reporting Trials of Electronic and Mobile Health Applications and Online Telehealth
IG: intervention group
IRB: institutional review board
ITT: intention-to-treat
MBSR: mindfulness-based stress reduction
PP: per-protocol
PSS-10: Perceived Stress Scale
RCT: randomized controlled trial
T0: time point 0
T1: time point 1
T2: time point 2
T3: time point 3
WCG: wait-list control group
