# Supplementary material for: Effects of a Mindfulness Intervention Comprising an App, Web-Based Workshops, and a Workbook on Perceived Stress Among Nurses and Nursing Trainees: Protocol for a Randomized Controlled Trial
Source: JMIR Res Protoc. 2022 Aug 2;11(8):e37195. doi: 10.2196/37195 (PMC9382546; doi:10.2196/37195)
Supplement: Multimedia Appendix 1 [file resprot_v11i8e37195_app1.pdf]

Einwilligungserklärung

zur Teilnahme an der schriftlichen Befragung im Rahmen des Projekts „Achtsamkeit in der akut-stationären Pflege“

Ich wurde über Inhalt und Ziel der Evaluation des Projekts „Achtsamkeit in der akut-stationären Pflege“ informiert. Die Evaluation wird unter der Leitung von Herrn Prof. Dr. Tobias Esch an der Universität Witten/Herdecke durchgeführt. Das Informationsschreiben für Teilnehmer*innen mit Hinweisen zum Datenschutz habe ich erhalten, gelesen und verstanden.

Durch meine Zustimmung zur Teilnahme an der Evaluation möchte ich das wissenschaftliche Vorhaben unterstützen. Ich stimme zu, dass ich zu vier Zeitpunkten zu einer Teilnahme an einer Online-Befragung per E-Mail eingeladen werde. Mir ist bekannt, dass alle Forschungsdaten anonymisiert ausgewertet werden. Eine Veröffentlichung erfolgt in anonymisierter Form. Meine Rechte in Bezug auf die Speicherung meiner personenbezogenen Daten nach der Datenschutzgrundverordnung (DSGVO) sind mir bekannt. Meine Teilnahme an der Evaluation ist freiwillig. Ich weiß, dass ich jederzeit mündlich oder schriftlich und ohne Angabe von Gründen meine Einwilligung zur Teilnahme zurückziehen kann, ohne dass mir daraus Nachteile entstehen.

Ich wurde umfassend über meine Rechte und auch über den zeitlichen Aufwand für mich als Teilnehmer*in informiert. Alle meine Fragen wurden verständlich und vollumfänglich beantwortet. Unter diesen Voraussetzungen willige ich in die Teilnahme an der schriftlichen Befragung ein.
