# Supplementary material for: Effects of a Mindfulness Intervention Comprising an App, Web-Based Workshops, and a Workbook on Perceived Stress Among Nurses and Nursing Trainees: Protocol for a Randomized Controlled Trial
Source: JMIR Res Protoc. 2022 Aug 2;11(8):e37195. doi: 10.2196/37195 (PMC9382546; doi:10.2196/37195)
Supplement: Multimedia Appendix 2 [file resprot_v11i8e37195_app2.docx]

| CONSORT-EHEALTH Checklist V1.6.2 Report  (based on CONSORT-EHEALTH V1.6), available at [[http](http://tinyurl.com/consort-ehealth-v1-6):[//tinyurl.com/consort-ehealth-v1-6].](http://tinyurl.com/consort-ehealth-v1-6) |
| --- |
|  |
| **Date completed** |
| 5/23/2022 13:09:46 |
| by |
| Simone Schönfeld |
| TITLE |
| Effects of a Mindfulness Intervention Comprising an App, Web-Based Workshops, and a Workbook on Perceived Stress Among Nurses and Nursing Trainees: Protocol for a Randomized Controlled Trial |
| **1a-i) Identify the mode of delivery in the title** |
| The app and the web-based workshops are delivered via the internet. |
| **1a-ii) Non-web-based components or important co-interventions in title** |
| The workbook is available only in printed form. |
| **1a-iii) Primary condition or target group in the title** |
| "[...] perceived stress among nurses and nursing trainees [...]" |
| ABSTRACT |
| **1b-i) Key features/functionalities/components of the intervention and comparator in the METHODS section of the ABSTRACT** |
| **"**The intervention group will receive a digitally supported mindfulness intervention, which will comprise an app, 2 web-based workshops, and a workbook, whereas the wait-list control group will be scheduled to receive the same intervention 14 weeks later." |
| **1b-ii) Level of human involvement in the METHODS section of the ABSTRACT** |
| "The two online workshops will be led by a certified Mindfulness Based Stress Reduction trainer. Nurses will use the app and the workbook independently." |
| **1b-iii) Open vs. closed, web-based (self-assessment) vs. face-to-face assessments in the METHODS section of the ABSTRACT** |
| "Recruitment will take place on the web and offline during the working hours of nurses and nursing trainees. [...] Self-report web-based surveys will be conducted on the web at baseline, at 10 weeks after allocation, at 24 weeks after allocation, and at 38 weeks after allocation." |
| **1b-iv) RESULTS section in abstract must contain use data** |
| Not applicable, data collection is still running. |
| **1b-v) CONCLUSIONS/DISCUSSION in abstract for negative trials** |
| Not applicable, data collection is still running. |
| INTRODUCTION |
| **2a-i) Problem and the type of system/solution** |
| See section "Need for effective stress management interventions for nurses" and "Requirements for Interventions in Acute Inpatient Care Settings". |
| **2a-ii) Scientific background, rationale: What is known about the (type of) system** |
| See section "Digitally supported mindfulness interventions". |
| **Does your paper address CONSORT subitem 2b?** |
| See section "Aims and Research Questions". |
| METHODS |
| **3a) CONSORT: Description of trial design (such as parallel, factorial) including allocation ratio** |
| See section "Design". For more details on randomization method see section "Assignment of Interventions: Sequence Generation". |
| **3b) CONSORT: Important changes to methods after trial commencement (such as eligibility criteria), with reasons** |
| Due date, there are no changes to methods after trial commencement. |
| **3b-i) Bug fixes, Downtimes, Content Changes** |
| Due date, there are no changes to methods after trial commencement. |
| **4a) CONSORT: Eligibility criteria for participants** |
| See section "Eligibility Criteria". |
| **4a-i) Computer / Internet literacy** |
| "Finally, the participants were required to have access to a smartphone." |
| **4a-ii) Open vs. closed, web-based vs. face-to-face assessments:** |
| See section "Recruitment". |
| **4a-iii) Information giving during recruitment** |
| "Informed consent was obtained from all participants before the start of each web-based survey." |
| **4b) CONSORT: Settings and locations where the data were collected** |
| "This study will be conducted at 4 hospitals and hospital-associated nursing schools in North Rhine-Westphalia, Germany. The number of nurses employed at each institution ranges from 40 to 1400. Two of the hospitals are acute care hospitals with emergency departments, whereas the other 2 hospitals specialize in pneumonology and cardiac surgery. The 2 nursing schools currently have a total of 280 and 380 enrolled nursing trainees." |
| **4b-i) Report if outcomes were (self-)assessed through online questionnaires** |
| "Data will be collected via a web-based data collection tool (LimeSurvey; LimeSurvey GmbH [31]) [...] All outcomes of interest consist of continuous variables based on self-report data." |
| **4b-ii) Report how institutional affiliations are displayed** |
| "Data will be collected via a web-based data collection tool (LimeSurvey; LimeSurvey GmbH [31]), provided by the study sponsor’s affiliated university. [comment: the logo of the university is visible] [...]. Furthermore, placebo effects and effects occurring because of participants’ expectations may occur with our study design. On the one hand, participants may experience some beneficial effects because of their anticipation of the upcoming intervention [52], which may be further increased by digitalization-specific expectations and participants’ trust in the study funder (a large health insurance company) [53]." |
| **5) CONSORT: Describe the interventions for each group with sufficient details to allow replication, including how and when they were actually administered** |
| **5-i) Mention names, credential, affiliations of the developers, sponsors, and owners** |
| "Neither of the authors has contributed to the development of the intervention." Managing directors authorized to represent the company: Jonas Leve, Manuel Ronnefeldt. |
| **5-ii) Describe the history/development process** |
| "App development started in 2014. Since then, the app has been one of the most frequently downloaded meditation applications in Germany; health insurance companies cover the costs associated with gaining access to specific app-based prevention courses that are otherwise only available via the premium version of the app. There is only one previous peer-reviewed evaluation among office workers indicating significant improvements in mindfulness, work engagement, job satisfaction, emotional exhaustion, emotional intelligence, innovation and creativity, and self-efficacy after using the app for 14 days [30]." For more information on content see See Table 2. |
| **5-iii) Revisions and updating** |
| "No substantial revisions to the app are planned, and the anticipated app updates are limited to minor bug fixes. The present intervention will use the app versions available in the Apple App Store and Google Play Store." |
| **5-iv) Quality assurance methods** |
| "No quality assurance methods have been planned to ensure the accuracy and quality of the information provided by the intervention provider." |
| **5-v) Ensure replicability by publishing the source code, and/or providing screenshots/screen-capture video, and/or providing flowcharts of the algorithms used** |
| See https://www.7mind.de/app |
| **5-vi) Digital preservation** |
| See https://www.7mind.de/app |
| **5-vii) Access** |
| "The intervention will be initiated by email. The email will contain the app access code, workbook pickup location at each hospital, and a list of proposed dates for web-based workshops. Participants will be given instructions on how to download the app from the app store and activate the code. The access code will allow access to the full app version, including meditations and education courses tailored to nurses for a 12-month period (Figure 1 [29]). The intervention will be free of charge for the participants." |
| **5-viii) Mode of delivery, features/functionalities/components of the intervention and comparator, and the theoretical framework** |
| See https://www.7mind.de/app |
| **5-ix) Describe use parameters** |
| "As regular practice (best case: daily) is considered a prerequisite for efficacy [18] [...] Participants will be instructed to meditate daily via the app, web-based workshops, and workbook." |
| **5-x) Clarify the level of human involvement** |
| "In-person interaction will occur only during the web-based workshops when participants will interact with the MBSR trainer and other group members. In case technical difficulties are encountered, information technology support will be available to the participants. [...] Nurses and nursing trainees will be invited to participate in 2 web-based workshops led by a certified MBSR trainer. The web-based workshops (approximately 60 minutes) will take place 1-2 weeks after app access has been granted. Up to 50 participants can take part in each web-based workshop." |
| **5-xi) Report any prompts/reminders used** |
| "No standardized prompts or reminders to meditate will be provided, and participants will be able to choose the frequency and duration of their meditation freely. Participants will have the option to choose whether to receive a weekly email newsletter from the intervention provider [...]. By receiving reminders to participate in the web-based surveys, participants are likely inadvertently reminded to interact with the app. As these reminders do not occur in real-world settings, the applicability of the findings from our RCT and others should be interpreted with caution." |
| **5-xii) Describe any co-interventions (incl. training/support)** |
| "The standardized multimodal stress management intervention comprises 3 components (an app, 2 web-based workshops, and a workbook). The web-based workshops and the workbooks are supplemental to daily app-based meditation." |
| **6a) CONSORT: Completely defined pre-specified primary and secondary outcome measures, including how and when they were assessed** |
| "Data collection for both groups will take place at 10, 24, and 38 weeks after allocation. In other words, data collection for each group will take place at 10 weeks (T1 for the IG and time point 2 [T2] for the WCG) and 24 weeks (T2 for the IG and time point 3 [T3] for the WCG) after intervention start. For individuals randomized to the IG, data will be collected at 38 weeks after the intervention starts (T3). [...] All outcomes of interest consist of continuous variables based on self-report data. Our primary outcome will be perceived stress measured using the 10-item Perceived Stress Scale (PSS-10) [32]. Secondary outcomes will include sense of happiness (Likert scale) [33], life satisfaction (L-1) [34], mindfulness (Five Facet Mindfulness Questionnaire) [35], well-being (World Health Organization-Five Well-Being Index) [36], self-care (Hamburg Self-Care Questionnaire; only pacing scale) [37], pain intensity (numerical rating scale 0-10) [38], work-related sense of coherence questionnaire [39], burnout (Copenhagen Burnout Inventory; work-related burnout scale and client-related burnout scale) [40], job satisfaction (Warr-Cook-Wall Scale [41], modified by Cooper et al [42]), and work engagement (Utrecht Work Engagement Scale) [43]." |
| **6a-i) Online questionnaires: describe if they were validated for online use and apply CHERRIES items to describe how the questionnaires were designed/deployed** |
| "To reduce detection bias, all questions were tested among members of the target group before the start of the study, and all outcome measures were validated in previous studies. Evaluations of the psychometric properties of our primary outcome, the PSS-10, in different countries report a Cronbach α of .78 to .91, with good test-retest reliability [57]." |
| **6a-ii) Describe whether and how “use” (including intensity of use/dosage) was defined/measured/monitored** |
| See section "Other Variables". |
| **6a-iii) Describe whether, how, and when qualitative feedback from participants was obtained** |
| "At the end of every survey, participants may enter feedback or additional comments into a text box." |
| **6b) CONSORT: Any changes to trial outcomes after the trial commenced, with reasons** |
| No changes. |
| **7a) CONSORT: How sample size was determined** |
| **7a-i) Describe whether and how expected attrition was taken into account when calculating the sample size** |
| See section "Sample Size". |
| **7b) CONSORT: When applicable, explanation of any interim analyses and stopping guidelines** |
| "No interim analyses are planned." |
| **8a) CONSORT: Method used to generate the random allocation sequence** |
| See section "Assignment of Interventions: Sequence Generation". |
| **8b) CONSORT: Type of randomisation; details of any restriction (such as blocking and block size)** |
| "Randomization was stratified by hospital and nursing school and job status (nurse or nursing trainee; 8 strata)." |
| **9) CONSORT: Mechanism used to implement the random allocation sequence (such as sequentially numbered containers), describing any steps taken to conceal the sequence until interventions were assigned** |
| See section "Assignment of Interventions: Sequence Generation". |
| **10) CONSORT: Who generated the random allocation sequence, who enrolled participants, and who assigned participants to interventions** |
| See section "Assignment of Interventions: Sequence Generation". |
| **11a) CONSORT: Blinding - If done, who was blinded after assignment to interventions (for example, participants, care providers, those assessing outcomes) and how** |
| **11a-i) Specify who was blinded, and who wasn’t** |
| Randomization and allocation was blinded. "Detection bias may occur as blinding of participants is not possible, given the nature of the study." |
| **11a-ii) Discuss e.g., whether participants knew which intervention was the “intervention of interest” and which one was the “comparator”** |
| "Although the study design is not communicated to the participants, the participants are to be likely aware of which group they have been randomized to. There may be a risk of social desirability as the effects will be evaluated based on subjective reports, including the frequency and intensity of digitally supported mindfulness intervention use, and no biological parameters will be collected." |
| **11b) CONSORT: If relevant, description of the similarity of interventions** |
| Not applicable, we will use wait list control groups. |
| **12a) CONSORT: Statistical methods used to compare groups for primary and secondary outcomes** |
| See section "Statistical Methods". |
| **12a-i) Imputation techniques to deal with attrition / missing values** |
| "Patterns of missing data will be assessed, and adequate data imputation techniques such as multiple imputation with SPSS Statistics (IBM) will be applied. Before doing so, we will assess the percentage of missing data. We will also use Little's Missing Completely At Random test to determine patterns of missing data (ie, missing completely at random vs not missing at random) [...]. Effectiveness will be evaluated using an intention-to-treat (ITT) approach; all randomized participants will be included in our analyses [45]. [...] To determine the reliability of the analyses for research questions 1 and 2, we will conduct sensitivity analyses for our primary and secondary outcomes. We will include only complete cases (complete data for T0, T1, T2, and T3)." |
| **12b) CONSORT: Methods for additional analyses, such as subgroup analyses and adjusted analyses** |
| No subgroup analyses are planned. "To ensure that our randomization has worked as intended, we will assess for between-group differences for relevant participant characteristics [...]. To ensure that our randomization has worked as intended, we will assess for between-group differences for relevant participant characteristics and, if needed, adjust the following analyses." |
| RESULTS |
| **13a) CONSORT: For each group, the numbers of participants who were randomly assigned, received intended treatment, and were analysed for the primary outcome** |
| Not applicable, data collection is still running. |
| **13b) CONSORT: For each group, losses and exclusions after randomisation, together with reasons** |
| Not applicable, data collection is still running. |
| **13b-i) Attrition diagram** |
| Not applicable, data collection is still running. |
| **14a) CONSORT: Dates defining the periods of recruitment and follow-up** |
| Not applicable, data collection is still running. |
| **14a-i) Indicate if critical “secular events” fell into the study period** |
| Not applicable, data collection is still running. |
| **14b) CONSORT: Why the trial ended or was stopped (early)** |
| Not applicable, data collection is still running. |
| **15) CONSORT: A table showing baseline demographic and clinical characteristics for each group** |
| Not applicable, data collection is still running. |
| **15-i) Report demographics associated with digital divide issues** |
| Not applicable, data collection is still running. |
| **16a) CONSORT: For each group, number of participants (denominator) included in each analysis and whether the analysis was by original assigned groups** |
| **16-i) Report multiple “denominators” and provide definitions** |
| Not applicable, data collection is still running. |
| **16-ii) Primary analysis should be intent-to-treat** |
| Not applicable, data collection is still running. |
| **17a) CONSORT: For each primary and secondary outcome, results for each group, and the estimated effect size and its precision (such as 95% confidence interval)** |
| Not applicable, data collection is still running. |
| **17a-i) Presentation of process outcomes such as metrics of use and intensity of use** |
| Not applicable, data collection is still running. |
| **17b) CONSORT: For binary outcomes, presentation of both absolute and relative effect sizes is recommended** |
| Not applicable, data collection is still running. |
| **18) CONSORT: Results of any other analyses performed, including subgroup analyses and adjusted analyses, distinguishing pre-specified from exploratory** |
| Not applicable, data collection is still running. |
| **18-i) Subgroup analysis of comparing only users** |
| Not applicable, data collection is still running. |
| **19) CONSORT: All important harms or unintended effects in each group** |
| Not applicable, data collection is still running. |
| **19-i) Include privacy breaches, technical problems** |
| Not applicable, data collection is still running. |
| **19-ii) Include qualitative feedback from participants or observations from staff/researchers** |
| Not applicable, data collection is still running. |
| DISCUSSION |
| **20) CONSORT: Trial limitations, addressing sources of potential bias, imprecision, multiplicity of analyses** |
| **20-i) Typical limitations in ehealth trials** |
| See section "Strengths and limitations". |
| **21) CONSORT: Generalizability (external validity, applicability) of the trial findings** |
| **21-i) Generalizability to other populations** |
| See section "Generalizability". |
| **21-ii) Discuss if there were elements in the RCT that would be different in a routine application setting** |
| See section "Generalizability". |
| **22) CONSORT: Interpretation consistent with results, balancing benefits and harms, and considering other relevant evidence** |
| **22-i) Restate study questions and summarize the answers suggested by the data, starting with primary outcomes and process outcomes (use)** |
| Not applicable, data collection is still running. |
| **22-ii) Highlight unanswered new questions, suggest future research** |
| Not applicable, data collection is still running. |
| Other information |
| **23) CONSORT: Registration number and name of trial registry** |
| "The study is registered as a clinical trial (DRKS00025997)." |
| **24) CONSORT: Where the full trial protocol can be accessed, if available** |
| Not applicable, this is the study protocol. |
| **25) CONSORT: Sources of funding and other support (such as supply of drugs), role of funders** |
| "This study was funded by BARMER, a German health insurance company. The funder’s responsibilities included initiating contact with participating clinics, approval of the final study design (randomized controlled trial with wait-list control groups), monitoring of study progress, and approval of manuscript submission for publication. The institute’s research team’s responsibilities included data collection, data management, data analyses, interpretation of results, and writing manuscripts. The authors would like to thank the cooperating hospitals and study participants for their support." |
| **X26-i) Comment on ethics committee approval** |
| See section "Research Ethics Approval and Amendments". |
| **x26-ii) Outline informed consent procedures** |
| "Informed consent was obtained from all participants before the start of each web-based survey. Informed consent materials are currently available only in German (Multimedia Appendix 1)." |
| **X26-iii) Safety and security procedures** |
| "Personal information about potential and enrolled participants will be collected only by members of the research team and cannot be accessed by other individuals. Personal information and survey data will be pseudonymized using an identification number (token). Only authorized study personnel will have access to any of the data associated with this study. The study funder reserves the right to share the anonymized data with other parties. [...] We will also seek to reduce the risk of social desirability by informing participants of the pseudonymization and anonymous publication of the data and by conducting data collection without in-person contact. In addition, we will not use participant data collected via the app but will allow participants to report data collected by the app. We refrained from tracking app use and the duration of use for privacy reasons. Not tracking app use may increase rapport, as participants will not feel monitored while using the app." |
| **X27-i) State the relation of the study team towards the system being evaluated** |
| "None declared. Neither of the authors has contributed to the development of the intervention." |
